# Ketogenic diet for mood disorders from animal models to clinical application

**DOI:** 10.1007/s00702-023-02620-x

**Published:** 2023-03-21

**Authors:** Ilya V. Smolensky, Kilian Zajac-Bakri, Peter Gass, Dragos Inta

**Affiliations:** 1 Department for Community Health, University of Fribourg, Fribourg, Switzerland; 2 Department of Biomedicine, University of Basel, Basel, Switzerland; 3 Department of Psychiatry and Psychotherapy, Medical Faculty Mannheim, Central Institute of Mental Health, Heidelberg University, Heidelberg, Germany

**Keywords:** Ketogenic diet, Sex differences, Depression, Bipolar disorder, Neuroinflammation, Neurogenesis

## Abstract

Mood disorders such as major depressive disorder (MDD) and bipolar disorder (BD) are often resistant to current pharmacological treatment. Therefore, various alternative therapeutic approaches including diets are, therefore, under investigation. Ketogenic diet (KD) is effective for treatment-resistant epilepsy and metabolic diseases, however, only a few clinical studies suggest its beneficial effect also for mental disorders. Animal models are a useful tool to uncover the underlying mechanisms of therapeutic effects. Women have a twice-higher prevalence of mood disorders but very little is known about sex differences in nutritional psychiatry. In this review, we aim to summarize current knowledge of the sex-specific effects of KD in mood disorders. Ketone bodies improve mitochondrial functions and suppress oxidative stress, inducing neuroprotective and anti-inflammatory effects which are both beneficial for mental health. Limited data also suggest KD-induced improvement of monoaminergic circuits and hypothalamus–pituitary–adrenal axis—the key pathophysiological pathways of mood disorders. Gut microbiome is an important mediator of the beneficial and detrimental effects of diet on brain functioning and mental health. Gut microbiota composition is affected in mood disorders but its role in the therapeutic effects of different diets, including KD, remains poorly understood. Still little is known about sex differences in the effects of KD on mental health as well as on metabolism and body weight. Some animal studies used both sexes but did not find differences in behavior, body weight loss or gut microbiota composition. More studies, both on a preclinical and clinical level, are needed to better understand sex-specific effects of KD on mental health.

## Introduction

High-fat low-carbohydrate ketogenic diet (KD) was originally introduced into clinical practice for the management of drug-resistant epilepsy nearly 100 years ago (Peterman 1925) and is still a very efficient treatment of epilepsy (Levy et al. 2012). Moreover, many studies have proven it as a powerful tool against obesity, metabolic and cardiovascular disorders (Zhu et al. 2022). The conventional macronutrient combination of KD is 4 g of fat to 1 g of protein and carbohydrates (4:1), resulting in almost 90% of calories coming from fats, 8% from protein and only 1–2% from carbohydrates. In contrast, for healthy individuals the recommended macronutrient profile is 20–30% of fat, 10–15% of protein and 45–65% of carbohydrates (Hee Seo et al. 2007). The main sources of fat in KD are lard, tallow, butter, fatty types of meat and fish, olive, coconut and avocado oil.

Besides epilepsy, metabolic and cardiovascular diseases growing amount of data suggest a beneficial effect of KD also in neurodegenerative diseases (Kashiwaya et al. 2000) and mental disorders, such as major depressive disorder (Brietzke et al. 2018), schizophrenia (Sarnyai et al. 2019) and autism-spectrum disorders (ASD) (Li et al. 2021).

Major depressive disorder (MDD) is a leading mental disorder affecting up to 300 million people worldwide, producing a huge burden on society’s wellbeing and the world economy (Liu et al. 2020). Bipolar disorder (BD) I and II affect around 1% of the world population and still represent one of the biggest challenges in psychiatry (Baldessarini et al. 2019). While current antidepressants do not entirely cure up to 40% of patients (Holtzheimer and Mayberg 2011), different non-pharmacological approaches are actively investigated for mental disorders which gave a rise to the new field of nutritional psychiatry (Sarris et al. 2015).

The mechanisms of the beneficial effects of diets in treating mood disorders are thought to include alleviation of neuroinflammation, hippocampal neurogenesis, neuroendocrine regulation and gut microbiota composition (Marx et al. 2020). On a molecular level, the cornerstone of these effects is an improved energy metabolism resulting in decreased free radicals production and cell death, which in turn reduces systemic inflammation (Marx et al. 2020).

Although no clinical trials have proven the efficacy of KD in MDD and BD patients, a beneficial effect was suggested by some case reports (Phelps et al. 2013; Danan et al. 2022). At the same time, the potential underlying mechanisms of the antidepressant effect of KD have been investigated in several preclinical studies (Huang et al. 2018; Guan et al. 2020) Here we summarize data on the postulated antidepressant effect of KD as well as its potential sex differences.

### Fatty acids and ketogenesis

Triglycerides and corresponding fatty acids are divided into three groups according to their length: short-chain fatty acids (SCFA), medium-chain FA (MCFA) and long-chain FA (LCFA) (Reviewed in (Lei et al. 2016)). SCFA (1–5 carbons: mainly acetate, butyrate and valproate) are produced by gut microbiota in the presence of dietary fibres and show neuroprotective and anti-inflammatory effects in animal models of mental and neurological disorders (Stilling et al. 2016). Saturated MCFA (6–12 carbons) are found predominantly in coconut oil and also in palm kernel oil, are absorbed and metabolized faster than LCFA (Hollis et al. 2018). LCFA includes mainly omega-3 polyunsaturated fatty acids with well-known anti-inflammatory and neuroprotective properties (Calder 2010) and omega-6 polyunsaturated fatty acids, first of all arachidonic acid, with pro-inflammatory activity and detrimental effects on a mental health (Simopoulos 2011).

Free fatty acids poorly cross the blood–brain barrier and thus do not provide energy for the brain unless they are converted into ketone bodies (Paoli et al. 2019). Ketogenesis takes place in mitochondria of the liver cells where fatty acids are metabolized to the ketone bodies β-hydroxybutyrate (BHB), acetate and acetoacetate (Fukao et al. 2004). The classic KD provides 60–80% of dietary energy from LCFA and not more than 40% from MCFA (Augustin et al. 2018). However, unlike LCFA, more lipid-soluble MCFA can cross cell and mitochondria membranes without transporters (Hamilton 1999) and are therefore considered the preferential source of ketone bodies (Seaton et al. 1986). MCFA-based diet, called alternative KD, comprises 60% lauric/octanoic (C12) acid and about 40% decanoic (C10) acid (Augustin et al. 2018). Meanwhile, one study reported that C8 MCFA supplement is about three times more ketogenic than C10 MCFA and about six times more ketogenic than C12 MCFA although the sample included only 9 participants (St-Pierre et al. 2019). Through monocarboxylic acid transporters ketone bodies pass the blood–brain barrier, enter the neurons and provide more energy per gram of oxygen than glucose (Hartman et al. 2007). Improved mitochondria function and energy supply along with antioxidant effects are thought to be the main mechanism of the beneficial effect of KD (Milder and Patel 2012). This in turn leads to neuroprotection and decreased neuronal death (Gasior et al. 2006) and, therefore, reduces brain and systemic inflammation (Koh et al. 2020).

### Treatment of mood disorders with KD

#### Clinical studies

No larger randomized clinical trials (RCT) have been performed for KD in any mental disorder up to date. Few studies are registered on clinicaltrials.gov for PTSD (NCT05415982), BD, psychotic disorders and schizophrenia (NCT03873922; NCT03935854), but have not reported their results yet. No RCTs of KD have been performed or started for MDD, while animal studies, case reports and a few small clinical trials suggest its beneficial effect on the mood. A recent small trial found improved depressive symptoms in 6 MDD patients and 12 BD patients after 3 weeks on KD (Danan et al. 2022). In healthy volunteers, KD improved mood and quality of life similarly (McClernon et al. 2007; Halyburton et al. 2007) or better (Yancy et al. 2009) than a low-fat diet, while another study found no effect on mental status in either diet (Iacovides et al. 2019). RCT of KD in epileptic patients found decreased anxiety and depressive symptoms independently of seizure control (IJff et al. 2016). Long-term mood stabilization was also reported in two female patients with BD II (Phelps et al. 2013).

Disturbed monoamine neurotransmission is a key mechanism of depression, schizophrenia, ADHD, ASD and other mental disorders (Hahn and Blakely 2002; Bortolato et al. 2008). Some data show that monoamine-related pathways could be targeted by KD. In epileptic children metabolites of dopamine and serotonin, but not noradrenaline were down-regulated in the cerebrospinal fluid after 3 months of KD (Dahlin et al. 2012).

Hypothalamus–pituitary–adrenal (HPA) axis dysregulation is a core symptom of mood and anxiety disorders with elevated cortisol level and disturbed feedback inhibition (Arborelius et al. 1999). Up to date, very few studies have investigated the effect of KD on the basal and stress-induced activity of the HPA axis. In healthy humans, KD did not change basal blood cortisol level as well as testosterone and thyroid hormones level (Volek et al. 2002; Volek and Sharman 2004).

Inflammation is also a common pathway for a wide range of mental disorders (Najjar et al. 2013) and a target of the beneficial effects of KD (Koh et al. 2020). In MDD patients, inflammation is thought to be connected with weight gain and obesity, thus distinguishing a metabolic and atypical subtype of depression (Lamers et al. 2013). The anti-inflammatory effect of KD was shown in both clinical and animal studies (see below). In obese patients, KD decreased blood TNFα and CRP levels while increasing blood Il-10 levels (Monda et al. 2020).

The gut microbiome is profoundly involved in the pathogenesis of various mental disorders, which are often associated with decreased diversity and altered composition of microbiota (Spichak et al. 2021; Nikolova et al. 2021). Gut microbiota is thought to mediate both the beneficial and negative effects of KD on metabolism and brain health (Attaye et al. 2022). KD increases the number of Bacteroidetes and *Akkermansia* in healthy sportsmen and obese patients while decreasing Actinobacteria (including Bifidobacteria) and Firmicutes (including Clostridiales) (Murtaza et al. 2019; Ang et al. 2020). Some authors argue that decline in gut Bifidobacteria and Lactobacillus could contribute to the possible detrimental effects of KD (Ang et al. 2020). Overall alpha-diversity was reported to be both increased by KD in obese patients (Gutiérrez-Repiso et al. 2019) or decreased by KD in children with epilepsy (Zhang et al. 2018).

#### Animal studies

Studies with KD or ketone body supplements in healthy rodents or translational stress models are summarized in Table 1. One study reported decreased basal anxiety in light–dark box (Hollis et al. 2018) but most of the studies with 2–4 weeks of KD did not find any effect on anxiety in elevated plus maze and light–dark box, depression-like behavior in forced swimming test and exploration in an open field (Murphy et al. 2005; Kasprowska-Liśkiewicz et al. 2017; Hollis et al. 2018; Ryan et al. 2018). One paper reported even increased anxiety and reduced exploration in an open field (Ling et al. 2019) which might, however, suggest a beneficial effect for ADHD (Murphy et al. 2005), although no clinical studies have been performed in patients yet. Meanwhile, long-term KD for 18 months ameliorated age-related memory decline in place avoidance test (Newman et al. 2017).

**Table 1:** Animal studies with ketogenic diet (KD) and ketone bodies supplement (KS)

Study	Animals	Intervention	Outcome
Healthy animals			
(Ari et al. 2017)	2 mo old Sprague–Dawley rats	Ketone ester (KE), BHB supplement (KS) or BHB + MCT (KSMCT)	KE, KS and KSMCT decreases anxiety in EPM and OFT
(Hollis et al. 2018)	2–3 mo old male Wistar rats	MCT-based KD for 15 days	Decreased anxiety in LDB Enhanced social competitive behavior in SDT No differences in depression in FST, sociability in TCT
(Kasprowska-Liśkiewicz et al. 2017)	4 wk old male Long-Evans rats	KD for 4 wk Ketone bodies for 3 days	Increased sociability in reciprocal SIT No effect on locomotion in OFT, anxiety in EPM, object memory in NOR No effect on sociability in reciprocal SIT
(Ling et al. 2019)	3 wk old Sprague–Dawley rats	Daily (KD) or every other day (KOD) for 1, 2 or 3 wk	KD (but not KOD) decreases locomotion and increases grooming in OFT after 3 weeks
(Maciejak et al. 2016)	Adult male Wistar rats	Single MCT intragastric administration	Elevated tryptophan metabolites in plasma and hippocampus 1.5 and 6 h after MCT intake
(Murphy et al. 2005)	Adult male Wistar rats	6.3:1 or 4:1 KD for 2 wk	No effect on locomotion, exploration and grooming in OFT
(Newman et al. 2017)	12–30 mo male C56/BL6 mice	1 wk ND: 1 wk KD for 18 mo	Prevents memory decline in PAT at 22 mo and NOR at 28 mo No effect on anxiety in EPM
(Ryan et al. 2018)	Adult male Long-Evans rats	KD for 3–4 wk MCT vs LCT by oral gavage	No effect on anxiety in EPM and despair in FST Elevated basal and restrain stress-induced CORT (but not ACTH), reduced thymus mass Increased CORT and ACTH elevation, c-Fos positive neurons in PVN
Models of depression and anxiety			
(Brownlow et al. 2017)	6–8 wk old male Sprague–Dawley rats	4 wk KD/KS → 5 wk KD/KS + restrain	Improved disturbances in spatial memory in MWM only by KD and in object memory in NOR by KD and KS No effect on stress-induced elevation of CORT and ACTH Attenuated hippocampal BDNF reduction by KD and KS
(Guan et al. 2020)	2–3 mo old male C57BL/6 J mice	LPS and R-SDS models of depression → 1–2 wk KD	Decreased despair in TST and FST, anhedonia in SPT, social avoidance in reciprocal SIT, microglia activation, neuronal excitability in the lateral habenula
(Huang et al. 2018)	8–10 wk old male C57BL/6 J mice	BHB for 3 days → LPS CUMS for 25 days → CUMS + BHB for 10 days	Increased microglia ramification, anti-inflammatory cytokines Prevents LPS and CUMS-induced despair in FST and TST, anhedonia in SPT
(Sahagun et al. 2019)	4 mo old male and female Long-Evans rats	CUMS + KD for 3wk	No effect of stress and diet on anxiety in EPM, despair in FST, IL-1β in serum, NPY and CRH in hypothalamus KD prevented CUMS-induced decreased in serum CORT in females
(Sahagun et al. 2021)	Adult male and female Sprague–Dawley rats	HFSD for 2 wk → ND or KD for 3 wk	HFSD-induced elevation of CORT in females was reduced by KD HFSD-induced decrease of CORT in males stayed unaffected by KD
(Yamanashi et al. 2017)	9–10 wk old male Sprague–Dawley rats	CUMS + BHB for 4–6 wk BHB injection → 1 h → acute immobilization stress	Prevented despair in FST, anhedonia in SPT, anxiety in EPM and NSF, decreased TNFɑ in hippocampus Decreased IL-1β and TNFɑ in hippocampus, serum CORT

*ACTH* adrenocorticotropic hormone, *CORT* corticosterone, *CUMS* chronic unpredictable mild stress, *EPM* elevated plus maze, *HFSD* high-fat high-sugar diet, *KD* ketogenic diet, *KS* ketone supplement (medium-chain triglycerides), *LPS* bacterial lipopolysaccharide, *FST* forced swimming test, *LCT* long-chain triglycerides, *LDB* light–dark box, *MCT* medium-chain triglycerides, *MWM* Morris water maze, *ND* normal diet, *NOR* new object recognition test, *NSF* novelty suppressed feeding test, *OFT* open field test, *PAT* place avoidance test, *PVN* paraventricular nucleus of hypothalamus, *R-SDS* repeated social defeat stress, *SDT* social dominance test, *SIT* social interaction test, *SPT* sucrose preference test, *TCT* three-chamber social preference test, *TST* tail suspension test

In stress models, KD showed a beneficial effect against behavioral disturbances. Chronic unpredictable mild stress for 4 weeks along with (Yamanashi et al. 2017) or followed (Huang et al. 2018) by BHB resulted in prevented depression-like behavior in the forced swimming test and sucrose preference test, anxiety in elevated plus maze and novelty suppressed feeding. Similar improvements in anhedonia and despair as well as in social avoidance were found in KD following repeated social defeat stress (Guan et al. 2020). Restrain stress-induced memory decline in the Morris water maze and new object recognition test was ameliorated by both KD and ketone bodies supplement (Brownlow et al. 2017). Along with behavioral improvements in stress models KD or ketone bodies suppressed inflammation and BDNF reduction in hippocampus (Brownlow et al. 2017; Yamanashi et al. 2017), microglia activation and neuronal excitability in the lateral habenula (Huang et al. 2018; Guan et al. 2020).

Improvement in social behavior were reported both in healthy animals (Kasprowska-Liśkiewicz et al. 2017; Hollis et al. 2018) and in mouse ASD models – maternal immune activation (Ruskin et al. 2017b), BTBR mice (Mychasiuk and Rho 2017; Ahn et al. 2020) and EL mice (Ruskin et al. 2017a). Given beforehand KD provides neuroprotection and reduces motor, mental and cognitive disturbances induced by traumatic brain injury in mice and rats (Brownlow et al. 2017; Salberg et al. 2019). Since BD is still difficult to model in animal experiments (Beyer and Freund 2017), no translational studies investigated the effects of KD in BD.

Few animal studies looked into the role of the monoamine system and HPA axis in the potentially beneficial effect of KD for mood disorders. Mice fed with KD for 3 weeks had significantly increased dopamine (but not serotonin) metabolism in the motor and somatosensory cortex, but not in the midbrain and basal ganglia. However, the total level of dopamine, serotonin and noradrenaline remained unchanged in all brain regions explored in this study (Church et al. 2014). In another study brain levels of tryptophan and its metabolite kynurenic acid were increased by MCFA-based KD (Maciejak et al. 2016). Dopamine β-hydroxylase knockout mice lacking noradrenaline showed no beneficial effect of KD against chemically induced seizures, suggesting the involvement of noradrenaline in the anti-epileptic effect of diet (Szot et al. 2001). Some animal studies report unchanged (Brownlow et al. 2017) or even increased basal and stress-induced activity of the HPA axis (Ryan et al. 2018).

Anti-inflammatory effect of KD was also shown in translational studies. In the animal stress models, BHB ameliorated stress-induced elevation of plasma Il-1β (Yamanashi et al. 2017), suppressed inflammatory microglia and activated neuroprotective microglia (Huang et al. 2018; Guan et al. 2020). KD-induced cognitive improvement was shown to be mediated by the expression of PPARγ-activated genes (Newman et al. 2017). Microglial PPARγ activation drives microglial reactivity (Fumagalli et al. 2018) and improves mitochondrial metabolism (Morris et al. 2020). Thus, PPARγ is thought to be a key pathway of the anti-inflammatory effect of KD. Along with microglia, astrocytes are also important mediators of the therapeutical effects of KD, as they were shown to change their shape and signaling upon the diet (Gzielo et al. 2019; Koppel et al. 2021).

The neuroprotective effect of KD is mediated by several mechanisms, such as increased expression of BDNF (Zhao et al. 2017) and anti-apoptotic factors (Cheng et al. 2007), as well as by decreased expression of pro-apoptotic factors (Noh et al. 2003) and suppressed release of cytochrome-c (Hu et al. 2009). Another molecular mechanism of the anti-inflammatory and neuroprotective effects of KD is improved energy metabolism in mitochondria, resulting in decreased oxidative stress. Mitochondria was shown to play an important role in the stress response and their impaired function can contribute to mental disorders (Daniels et al. 2020). Ketone bodies increase the expression of bioenergetic enzymes (Bough et al. 2006), glutathione levels and glutathione peroxidase activity (Ziegler et al. 2003; Jarrett et al. 2008). Improved mitochondria function was shown to mediate the beneficial effect of KD in animal models of ASD (Ahn et al. 2020), epilepsy (Bough et al. 2006) and amyotrophic lateral sclerosis (Zhao et al. 2006).

Similarly to human patients KD increases the number of Bacteroidetes and *Akkermansia* in rodents while decreasing Actinobacteria (including Bifidobacteria) and Firmicutes (including Clostridiales) (Ma et al. 2018; Ang et al. 2020; Park et al. 2020). *Akkermansia*-based probiotics showed antidepressant-like properties in the animal stress model (Ding et al. 2021) suggesting also its potential role in the antidepressant effect of KD. Despite a general reduction in the number of gut Firmicutes, the number of Lactobacillus was reported by different studies to either decrease or increase (Ma et al. 2018; Ang et al. 2020; Park et al. 2020). In animal studies, alpha-diversity was also reported to be increased (Yue et al. 2021) or decreased (Ma et al. 2018; Olson et al. 2018). Still very little is known about the role of the gut microbiome in the treatment of mental disorders with KD. One work showed its role in a mouse ASD model (Newell et al. 2016), while KD-induced improvement in other psychiatric disorders have not been directly associated with the gut microbiome.

Despite its impressive beneficial effects on different disorders, KD has also a number of disadvantages. It is not only hard to follow (Cavaleri and Bashar 2018) but it is also associated with some harmful side effects including growth retardation, nephrolithiasis, hyperlipidemia and atherogenesis (Kang et al. 2004; Hartman and Vining 2007). One key therapeutic goal is therefore to try to replace the KD and its strict requirements for adherence with dietary supplements that can produce sustained ketosis and mimic the effects of the ketogenic diet. Exogenous ketone body supplements considered as a future “ketogenic diet in a pill” showed beneficial effects in patients with obesity (Walsh et al. 2021) and mild cognitive impairment (Roy et al. 2021). Animal studies of anxiety, mood and cognitive disturbances also reported promising results of ketone supplementation (Ari et al. 2017, 2020; Brownlow et al. 2017; Huang et al. 2018; Shcherbakova et al. 2023).

#### Sex differences in the effects of the ketogenic diet

Many studies have found sex differences in the effect of diets on various metabolic and cardiovascular diseases, while very few described such sex-specific effects of nutrition in mental disorders. KD seems to reduce body weight, fat mass and γ-glutamyl transferase (marker of non-alcoholic fatty liver disease) better in men compared to pre-menopausal, but not post-menopausal women (Volek et al. 2004; D’Abbondanza et al. 2020). Authors suggest that one reason could be higher levels of more metabolically active visceral adipose tissue in men compared to women (Gerdts and Regitz-Zagrosek 2019). Another study also reported a higher decrease in body weight and fat mass in obese men on KD although the blood BHB level was greater in obese women after 9 weeks of KD. Diet-induced decrease in GLP-1 but not in other appetite hormones was found to be stronger in women but its role in the overall effect of KD remains unclear (Lyngstad et al. 2019).

One mice study reported body weight gain and glucose tolerance in females, but weight loss in males (Cochran et al. 2018). Higher KD-induce elevation in blood ketone bodies and attenuation of blood glucose level were found in young female mice and rats (Dai et al. 2017; Cochran et al. 2018), while from 14 to 17^th^ months of life these changes were stronger in males (Kovács et al. 2021). Together with differences between pre- and post-menopausal women (Volek et al. 2004; D’Abbondanza et al. 2020) these data show age-dependent sex differences in the effect of KD.

A recent study compared C8 and C10 MCFA-induced ketogenesis during aerobic exercise in middle-aged men and women with average body mass index and low physical activity. Carbohydrate oxidation was enhanced only in women on both diets, while in men on the C8 diet carbohydrate oxidation was suppressed along with enhanced fat oxidation (Nosaka et al. 2021). As no differences between C8 and C10 MCFA were found in women, C8 MCFA might be a more effective source of ketone bodies than C10 (St-Pierre et al. 2019) only in men but not in women. But given the small sample of only 9 participants (St-Pierre et al. 2019) these results should be taken with caution.

Rats fed with a high-fat high-sugar diet for 12 weeks showed opposite changes in HPA axis activity with decreased corticosterone level in males but increased level in females (Sahagun et al. 2021). Switching to KD partially reduced corticosterone level in females while no changes were observed in males. KD-induced weight loss was found in both sexes, but it correlated with plasma ketone bodies only in females (Sahagun et al. 2021). Given that improved mitochondrial function is thought to be a key mechanism of KD effects, it is worth to mention that in a rat model of traumatic brain injury, as well as in a control group, females demonstrated higher KD-induced increase of mitochondrial optic atrophy-1 (*Opa1)* gene expression (Salberg et al. 2019).

Sex-specific effects of KD on mental health were evaluated almost only in animal studies, although one study with obese volunteers did not find any sex differences in KD-induced changes in mood and cognitive function (Halyburton et al. 2007). Stress-induced loss in body weight was found in both male and female rats, and in both groups it was prevented by KD (Sahagun et al. 2019). Stress-induced anxiety appeared only in females while corticosterone level in females decreases upon stress chronic mild stress which makes it difficult to compare the protective effects of KD between sexes. However, in females anxiety and corticosterone level remained unchanged under stress upon KD with no effect of diet in males (Sahagun et al. 2019). High-fat diet-induced obesity was accompanied by increased anxiety only in male mice and only in males diet enhanced further stress-related reduction of locomotion (Bridgewater et al. 2017). Despite some data that KD enhances neurogenesis in hippocampus (Benjamin et al. 2017), one study compared its effects between sexes and found no increase in either males or females (Strandberg et al. 2008). Higher rate of inflammation in women was suggested to be a reason why female depression patients are 2–3 times more likely to have atypical symptoms such as increased appetite and body weight (Eid et al. 2019). Since inflammation is a key target of KD it might be expected that its antidepressant effect is higher in women, however, no studies have confirmed this assumption so far.

Still very little is known about sex differences in gut microbiota in general (Reviewed in (Jaggar et al. 2020)) and even less about their role in mental health and dietary interventions (Reviewed in (Manosso et al. 2021)). Pre-menopausal but not middle-aged women seem to have higher bacterial α-diversity (Jaggar et al. 2020) and lower gut permeability which are both thought to be bidirectionally regulated by sex hormones (Audet 2019). Firmicutes-to-Bacteroidetes ratio is well-known to be increased in a number of mental disorders including depression and schizophrenia (Nikolova et al. 2021). Whether this ratio changes differently in male and female psychiatric patients remains unknown, while only one study reported different gut microbiota changes in men and women with depression (Chen et al. 2018). Correlation with depressive symptoms was also found for different bacterial genera in males and females in this study. Similarly, early-life adversity in mice resulted in sex-specific behavioral and gut microbiota changes (Rincel et al. 2019).

Sex differences in diet-induced changes in gut microbiota were reported only in a few studies with polyphenol (Most et al. 2017) or oligofructose supplementation (Shastri et al. 2015) and low-fat diet (Santos-Marcos et al. 2019) but not in KD. One study which showed an important role of gut microbiota in the antiepileptic effect of KD compared both sexes but did not find any differences (Olson et al. 2018). Higher overall abundance of SCFA-producing bacteria was shown in female mice (Hongchang et al. 2019) and can be important for sex differences in dietary effects. In summary, despite some data about sex differences in diet- and mental disorders-associated changes in gut microbiota, no sex-specific effects of KD in depression have been published.

## Conclusion

Data from experimental animal models and small clinical studies suggest that the ketogenic diet may have beneficial effects both in MDD patients and in the healthy population. Its repeatedly proven neuroprotective and anti-inflammatory effect is thought to be a key mechanism of its potential antidepressant effect. Moreover, key pathways of MDD pathogenesis, such as HPA axis hyperactivity and brain monoamine circuit disturbances have also been shown to be improved by KD in animal stress models. Recent animal studies using depression models showed synergistic effects of antidepressants combined with omega-3-rich fish oil (Paula Farias Waltrick et al. 2022) and caloric restriction (Haritov and Tivcheva 2020). High-fat Western diet, on contrary, diminished the efficacy of antidepressant treatment (Sial et al. 2021). Future studies might shed more light on the interaction between pharmacological treatment and nutritional interventions, particularly KD. Very little is still known about sex differences in the overall effects of KD on body weight, metabolism and mental health. Studies in which both sexes have been used report contradictory results. Therefore, it is too early to judge whether KD acts differently in male and female MDD patients. More data are needed for valid conclusions. To address these questions, future preclinical and clinical studies using KD in psychiatry should include both sexes.

## Abbreviations

ADHD: Attention deficit and hyperactivity disorder
ASD: Autism-spectrum disorder
BHB: β-Hydroxybutyrate
BD: Bipolar disorder
HPA axis: Hypothalamus–pituitary–adrenal axis
KD: Ketogenic diet
LCFA: Long-chain fatty acids
MCFA: Medium-chain fatty acids
MDD: Major depressive disorder
RCT: Randomized controlled trial
SCFA: Short-chain fatty acids
